# On the rising incidence of early breast development: puberty as an adaptive escape from ectopic adiposity in mismatch girls

**DOI:** 10.1530/EJE-21-0287

**Published:** 2021-05-05

**Authors:** Francis de Zegher, Lourdes Ibáňez

**Affiliations:** 1 University of Leuven, University of Leuven, Leuven, Belgium; 2 University of Barcelona, Barcelona, Spain

Over the past decades, there has been a worldwide trend towards younger ages of pubertal onset in girls (1). Overall, this trend has been estimated at 3 months per decade (1) but some regions are experiencing faster transitions, in parallel with rapid gains in body weight. In Korea, for example, the prevalence of precocious puberty in girls has increased >10-fold within a single generation (2). Such alarming rises are not readily attributable to exposures to endocrine disruptors (3) and are still under active investigation.

One of the proposed hypotheses is that an earlier/faster maturation in girls is the phenotypic expression of an adaptive mechanism whereby girls attempt to escape from ectopic adiposity which, in turn, ensues from a mismatch between reduced prenatal weight gain (with reduced s.c. adipogenesis, thus a reduced capacity for safe lipid storage) and augmented postnatal weight gain (with augmented lipogenesis, and thus an augmented need for lipid storage) (4). This hypothesis builds upon the more general mismatch concept that offers an explanation for a variety of recent trends and emerging phenotypes (5). For example, the mismatch between reduced prenatal weight gain and augmented postnatal weight gain contributes to explain the trends towards higher blood pressures in early childhood (6), towards exaggerated adrenarche (higher concentrations of circulating DHEAS sometimes eliciting a precocious pubarche) (7), towards younger ages at menarche (8), and towards higher incidences of polycystic ovary syndrome (9). In the context of precocious puberty, the presence or absence of such a mismatch can readily be estimated by calculating the upward change in Z-score (or centile) between birthweight-for-gestational-age and BMI-at-onset-of-puberty (4, 10). Hitherto, however, this mismatch hypothesis has remained untested as a potential explanation for the increasing incidence of precocious puberty.

In a recent issue of the European Journal of Endocrinology, Harbulot *et al*. (11) reported how they performed the first test of the mismatch hypothesis, by analysing their single-centre cohort of girls with isolated variants of central precocious puberty (*n* = 319) in an unprecedented way: they distinguished three subgroups and calculated the Z-scores of birthweight-for-gestational-age and BMI-at-diagnosis-of-central-precocious-puberty in each subgroup. Their findings (Fig. 1) endorse the mismatch concept since the three subgroups ('familial', 'sporadic', and 'adopted') tended to differ in birthweight (average centiles 50, 34, and 10, respectively) and also in BMI-at-diagnosis (centiles 96, 90, and 76), so that the upward changes between birth and puberty amounted on average to 46, 56, and 66 centiles. These marked increments are likely to be underestimated since the early (= pre-diagnosis) phase of central precocious puberty is characterised by an acceleration of height gain that often has a lowering effect on BMI; it is by growing faster that girls can escape from ectopic adiposity.

**Figure 1:**
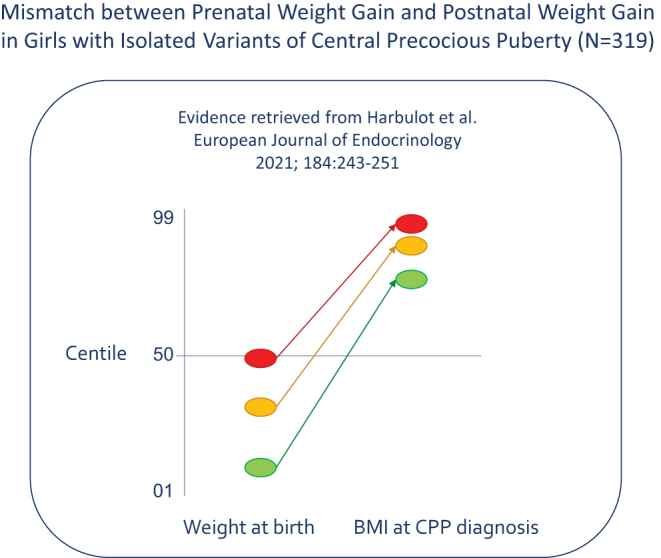
Mismatch between prenatal weight gain and postnatal weight gain in girls with isolated variants of central precocious puberty (CPP; *n* = 319). Weight at birth is expressed in centiles for sex and gestational age. BMI at diagnosis of CPP is expressed in centiles for sex and chronological age. The authors of the original paper (11) distinguished three subgroups of girls with isolated CPP. In the 'familial' subgroup (red), the average centile rose from 50 (weight at birth) to 96 (BMI at diagnosis of CPP), for an upward mismatch of 46 centiles. In the 'sporadic' subgroup (orange), the average centile rose from 34 to 90, for an upward mismatch of 56 centiles. In the 'adopted' subgroup (green), the average centile rose from 10 to 76, for an upward mismatch of 66 centiles. Marked mismatches are known to be associated with insulin resistance and ectopic fat in girls aged 8 years (10).

In conclusion, a vast majority of girls with isolated variants of central precocious puberty were found to have experienced an upward mismatch between prenatal and postnatal weight gain. The clinical data of Harbulot * et al.* (11) allowed us to infer this simple message which, however, implies that treatment in most girls with precocious puberty should aim not only at silencing the gonadotropic axis but also at reducing weight gain and/or ectopic fat. Central precocious puberty in girls should henceforth be viewed as possibly reflecting the activation of the gonadotropic axis in a homeostatic and evolutionarily conserved attempt to escape from ectopic adiposity.

## Declaration of interest

The authors declare that there is no conflict of interest that could be perceived as prejudicing the impartiality of this article.

## Funding

This work did not receive any specific grant from any funding agency in the public, commercial, or not-for-profit sector.
